# LDP alleviates TKI-induced proteinuria through reversing the expression of RelA in renal tissues

**DOI:** 10.3389/fmed.2023.1095344

**Published:** 2023-01-19

**Authors:** Zhou Fu, Su Zhang, Xiaoying Gu, Tao Guan, Chengmeng Wang, Jiaqi Zhang, Yun Wang, Hua Guo, Lu Wang, Ti Zhang

**Affiliations:** ^1^Key Laboratory of Cancer Prevention and Therapy, Department of Hepatobiliary Surgery, National Clinical Research Center for Cancer, Tianjin Medical University Cancer Institute and Hospital, Tianjin, China; ^2^Key Laboratory of Cancer Prevention and Therapy, Department of Gynecologic Oncology, National Clinical Research Center for Cancer, Tianjin Medical University Cancer Institute and Hospital, Tianjin, China; ^3^Department of Cell Biology and Medical Genetics, School of Basic Medical Science, Shanxi Medical University, Jinzhong, China; ^4^Key Laboratory of Cancer Prevention and Therapy, Department of Tumor Cell Biology, National Clinical Research Center for Cancer, Tianjin Medical University Cancer Institute and Hospital, Tianjin, China; ^5^Department of Hepatic Surgery, Fudan University Shanghai Cancer Center, Shanghai, China; ^6^Department of Oncology, Shanghai Medical College, Fudan University, Shanghai, China

**Keywords:** proteinuria, tyrosine kinase inhibitors, Liuwei Dihuang Pill, tumor, hepatocellular carcinoma

## Abstract

Tyrosine kinase inhibitors (TKIs), as an important tumor therapy, can induce severe proteinuria that significantly affects anti-tumor therapy. Existing therapies against proteinuria induced by other etiologies are currently ineffective for TKI-induced proteinuria. It has been shown that various types of proteinuria are related to podocyte damage caused by changes in the RelA signaling pathway. Our experiments confirmed that TKIs activate the renal RelA signaling pathway, and induce death of podocytes and destruction of the glomerular filtration barrier. Here we found that Liuwei Dihuang Pill (LDP) attenuated the inflammatory injury of podocytes through inhibiting activation of RelA, and subsequently relieved TKI-related proteinuria and prevented the progression of TMA and FSGS. Our finding indicated that LDP may be effective for the treatment of TKI-induced proteinuria, which is clinically significant.

## Introduction

Tyrosine kinase inhibitors (TKIs) significantly improve the survival of terminal cancer patients while the targeted drugs can cause some non-negligible adverse events, including hypertension, proteinuria, hand-foot syndrome, diarrhea, bleeding, etc. Except for proteinuria, most of these adverse events can be effectively controlled. In clinical trials of sorafenib, varying degrees of proteinuria occurred in 21–63% of patients (1–3), compared with 47.7% in apatinib users (4). According to two recently published phase II clinical trials, the incidence of proteinuria was as high as 58% (5) and 83% (6) for regorafenib and lenvatinib, respectively. For patients who were prescribed lenvatinib, the most frequent adverse event that resulted in decreased medications was proteinuria (52%) (6). Due to its high incidence and significant influence on the tumor therapy of proteinuria, it is urgent to solve the issue. Currently, Angiotensin-converting enzyme inhibitors (ACEIs) are commonly used for proteinuria induced by other causes, but our study proved that ACEIs cannot alleviate proteinuria caused by TKIs, and even weaken the anti-tumor effect of TKIs (7). There is no effective treatment for proteinuria induced by TKIs. Thus development of new treatment to alleviate TKIs-related proteinuria has become a priority, and the key is to deeply explore the pathogenesis of TKIs-related proteinuria.

Numerous studies on proteinuric nephropathy have shown that an intact glomerular filtration barrier is essential for the maintenance of normal renal function and is also associated with the onset of proteinuria. Basement membrane, fenestrated capillary endothelium and podocytes form this barrier together (8). With a complex structure, podocytes are highly differentiated cells that line the outside of the glomerular capillaries, which are featured by the protuberance and Bowman’s capsule. Normal podocyte structure is essential for normal glomerular permeability (9). Most proteinuric kidney disease manifests as fusion or loss of podocyte foot processes. A study including patients with diabetic nephropathy showed a significant correlation between podocyte loss and increased albumin excretion rate (AER). Moreover, AER was directly related to the foot process width, and the podocyte number was the strongest predictor of proteinuria progression (10). Podocyte-specific vascular endothelial growth factor-A (VEGFA) knockout mice developed marked proteinuria and the kidneys showed thrombotic microangiopathy (TMA), a lesion characterized by fibrin platelet thrombus formation. Consistently, bevacizumab resulted in the same renal pathological changes. Our previous study showed that TKIs-treated mouse kidneys exhibited two pathological types: focal segmental glomerulosclerosis (FSGS) lesions characterized by fusion of podocyte foot processes and TMA characterized by endothelial damage (7). It is suggested that TKIs-associated proteinuric nephropathy may be mainly associated with endothelial cells and podocytes, but it is still unclear which cell damage plays a key role. We have previously confirmed that TKIs can cause endothelial cell damage (7), and this study focuses on podocytes.

Mammalian NF-κB signaling pathway is an important intracellular transcription factor system that is induced by extracellular stimuli from different sources. Nuclear translocation of dimeric Rel proteins can serve as a hallmark of NF-κB activation, which regulate hundreds of NF-κB-dependent genes involved in inflammation, immunity, apoptosis, cell proliferation, and differentiation. Increasing evidence shows that the activation of NF-κB is a key response in renal disease. The NF-κB p65 (RelA) staining score was positively correlated with the severity of proteinuria (11), and binds to the cmaf inducing protein (c-mip) promoter and inhibits its transcriptional activity. Estrada et al. demonstrated increased activation of NF-κB signaling and translocation of RelA to the nucleus in glomerular endothelial cells and podocytes when a vascular endothelial growth factor (VEGF) inhibitor was used. Moreover, RelA was retained in the cytoplasm when a vascular endothelial growth factor receptor (VEGFR) inhibitor was used, and c-mip overexpression in podocytes led to cytoskeletal changes and nephrotic syndrome (12). Izzedine et al. confirmed that TMA lesions showed high enrichment of RelA in endothelial cells and podocyte nuclei, and c-mip was almost not detected, while MCN/FSGS showed high enrichment of c-mip, but RelA was almost not detected (13). Our study demonstrated that treatment of tumor-bearing mouse models of hepatocellular carcinoma with different TKIs such as apatinib, lenvatinib, and regorafenib resulted in increased activation of RelA signaling in glomerular endothelial cells and podocytes.

Liuwei Dihuang Pill (LDP) is an ancient Chinese medicine formula whose effects on chronic inflammation, oxidative stress, and diabetes-related kidney disease have been confirmed (14). Previous studies have confirmed that LDP can reduce proteinuria in diabetic nephropathy by inhibiting NF-κB, MAPK, TGF-β/SMADS, and other signaling pathways, slow down renal fibrosis, and protect renal mesangial cells (15). In addition to reducing proteinuria, LDP can also inhibit malignant tumors. Zheng et al. confirmed that LDP can inhibit the metastasis of breast cancer by inhibiting the expression of TCF-1, β-catenin, cyclin-D1 (16). In addition, LDP can also suppress the development of gastrointestinal tumors (17, 18). This study investigated the effect of LDP on TKIs-induced proteinuria and the possible mechanism, and it was demonstrated that both the function and pathological changes of kidneys in mice treated with TKIs were improved after LDP treatment. Our study showed that LDP can protect podocytes from the inflammatory injury induced by RelA signaling pathway, thereby alleviating proteinuria and potentiating the anti-cancer effect of apatinib.

## Materials and methods

### Materials

Liuwei Dihuang Pill (LDP) was purchased from Peking Tongrentang Pharmaceutical Co., Ltd. (batch number: Z19993608, weighed 1.44 g/8 pills). It is constituted of Rehmannia glutinosa (Gaertn.) DC., Paeonia × suffruticosa Andrews., Dioscorea oppositifolia L. [Dioscoreaceae]., Smilax glabra Roxb. [Smilacaceae]., Alisma plantago-aquatica subsp. orientale (Sam.) Sam. [Alismataceae]., Cornus officinalis Siebold & Zucc. Regorafenib (BAY 73-4506), and Lenvatinib (E7080) was purchased from Selleck. Apatinib (HY-13342S) was purchased from MCE.

### Cell lines

Two hepatocellular carcinoma cell lines were donated by the Liver Cancer Institute, Zhong Shan Hospital in Shanghai, MHCC-97H (human) and Hep1-6 (mouse), were used in our study. The cells were cultured in the DMEM culture medium (Corning 10-013-CV, USA) in the condition of 37°C, 5% CO_2_ and the culture medium was mixed with fetal bovine serum (10%; Corning 35-056-CM, USA). Besides, streptomycin (10 mg/ml; Corning 61-088-RM, USA) and penicillin (10 U/ml; Corning 30-001-CI, USA) were also added into the medium to resist bacterial attack. The medium was tested by Mycoplasma Detection Kit to exclude the mycoplasma pollution.

### Tumor models of mice

Male C57BL/6 and BALB/c nude mice (age, 4 weeks) were used to establish the tumor models of mice (*n* ≤ 6 in each cage), and the mice were raised in Experimental Animal Center, Tianjin Medical University Cancer Institute and Hospital. 100 μl PBS suspended 1 × 10^7^ MHCC-97H cells were injected into subcutaneous tissues at the lateral margin of the forearm in BALB/c mice, and 1 × 10^7^ Hep1-6 cells into C57BL/6 mice (7). After the tumors were transplanted into the mice, they were randomly divided into four groups: control, LDP, apatinib, LDP + apatinib; or two groups: control, TKIs.

LDP or TKIs (apatinib, regorafenib, and lenvatinib) were administrated orally into the tumor-bearing mice based on grouping, once daily. Glucose solution (5%, w/v) and carboxymethyl cellulose (0.5%, w/v) were used to dissolve apatinib and lenvatinib, with LDP and regorafenib dissolved in transcutol/cremophor/sodium chloride (ratio, 1:1:8). Tumor volume changes were measured three times a week, including the corresponding perpendicular (B), the largest diameter (A), and the perpendicular to the plane of B and A (C). The final volume was calculated as (A × B × C)/2 (7). All animal experiments were approved by the Institutional Animal Care and Use Committee of Tianjin Medical University Cancer Institute and Hospital (Approval No. AE-2021001).

### Collection of tumor tissues and organs

The mice were sacrificed according to ethical requirements. After the kidneys and tumor tissues were removed and measured, 4% (wt/vol) paraformaldehyde (PFA) was used to fix the tissues and organs overnight, which were then washed by PBS and embedded by paraffin.

### Analysis of urine

Mouse 24-h urine was collected by a metabolic cage weekly. 10 μl of urine were taken for Coomassie staining, and gross albuminuria was detected by SDS-PAGE gel in each group. The ELISA kit (Bethine, USA) was used for quantitative analysis. The colorimetric assay kit (Exocell, USA) was used for determination of urine creatinine according to the instruction.

### Immunohistochemistry

RelA and c-mip immunohistochemical staining was performed in the laboratory of Tianjin Medical University Cancer Institute and Hospital. The renal tissue slides were incubated with hydrogen peroxide (3%) for 10 min, and then 1:200 RelA primary antibody (Abcam, ab32536, USA) or 1:200 c-mip primary antibody (Proteintech, 12851-1-AP, China) was added separately and incubated with the samples for another 40 min under room temperature. After that, the tissues were washed by buffer, and smeared with a mouse/rabbit linker (sc2357; provided by Santa Cruz, USA) for 10 min. They were then incubated with the secondary antibody for 20 min. Negative and positive controls were treated similarly. The scoring criteria for staining results were as follows: 0, no staining; 1, weak staining; 2, moderate staining; 3, strong staining; 0, positive staining area of 0–10%; 1, positive staining area of 10–25%; 2, positive staining area of 25–50%; 3, positive staining area of 50–100%. IHC score = positive staining × positive staining area (e.g., 0, no staining; 1, positive staining area of 10–25%; IHC score = 0 × 1 = 0).

### Western blot

ProteinExt^®^ Mammalian Total Protein Extraction Kit (TransGen Biotech, China) was applied to extract the protein in tumor and renal tissues based on the instructions, and anti-VEGFA (ab1316; provided by Abcam, USA), VEGFR2 (ab39256; provided by Abcam, USA), β-actin (ab179467; purchased from Abcam, USA), P-VEGFR2 (ab5473; provided by Abcam, USA), RelA (ab32536; Abcam, USA) and c-mip (12851-1-AP; proteintech, China) primary antibodies as well as mouse anti-rabbit secondary antibodies (#7076, #7074; purchased from Cell Signaling Technology, USA) were used for Western blot. Gel-Pro Analyzer (provided by Media Cybernetics, MD, USA; Version 4.0) was used for protein analysis.

### Real-time PCR

For mRNA isolation, fresh mouse kidney pieces were pulverized in an Eppendorf Tube (sterilized) and dissolved in 1 ml Trizol (Ambion, USA, 380502). Spectrophotometry was used to measure the concentration of the collected RNA. Based on a quantitative real-time PCR (RT-PCR) kit (Takara, Japan), cDNA was synthesized by reverse transcription of the isolated RNA. GAPDH was used as control for normalization. The mouse IL-17A, IFN-γ, IL-6, IL-4, IL-10 and GAPDH primers were synthesized by BGI Bio-Solutions (Beijing, China) Co., Ltd., and the sequences of the primers used are listed in Table 1.

**TABLE 1:** Primers used in real-time PCR.

Gene	Forward	Reverse
IFN-γ	GCCACGGCACAGTCATTGA	TGCTGATGGCCTGATTGTCTT
IL-6	CTGCAAGAGACTTCCATCCA	AGTGGTATAGACAGGTCTGTTGG
IL-4	GGTCTCAACCCCCAGCTAGT	GCCGATGATCTCTCTCAAGTGAT
IL17A	TCAGCGTGTCCAAACACTGAG	CGCCAAGGGAGTTAAAGACTT
IL-10	CTTACTGACTGGCATGAGGATC	GCAGCTCTAGGAGCATGTGG
GAPDH	TGGCCTTCCGTGTTCCTAC	GAGTTGCTGTTGAAGTCGCA

### Transmission electron microscopy

Pre-cooled PBS (pH 7.4) was used to wash the kidneys, and renal tissues were incubated with 0.1M PBS (pH 7.4) and glutaraldehyde (2.5%) overnight. The treated tissues were cut into slides of 50 μm in thickness with a vibrating microtome, fixed in 1% osmium tetroxide for 1 h, dehydrated in graded ethanol, embedded in epoxy resin and polymerized at 80°C for 24 h. After that, the slides were cut into ultra-thin sections (100 nm) with uranyl acetate and lead citrate for further observation under a JEM2000EX transmission electron microscope (TEM; JEOL, Tokyo, Japan).

### Immunofluorescence staining

Frozen sections of mice tissues made by the Pathology Department in our hospital were incubated with polyclonal primary antibodies against WT-1 (Abcam, ab267377, USA) overnight and goat anti-rabbit secondary antibodies (Abcam, ab150081, USA) IgG H&L (Alexa Fluor^®^ 488) for 1 h. After PBS washing for 3 times, 1 μg/ml DAPI was used to stain the sections and Dako Fluorescence Mounting Medium was used to mount the sections, which were stored at four centigrade degree after covered by a glass. Inverted fluorescence microscopy (DMI6000B; provided by Leica, USA) was performed for imaging, and ImageJ software was used for analysis.

### Histological evaluation

According to standard techniques, kidney tissues were sectioned at a thickness of 3 μm, and stained with Masson’s trichrome staining kits (Sigma, USA), Periodic Acid Schiff (PAS) and hematoxylin and eosin (H&E). The mean glomerular volume area was calculated based on the average volume of 30 glomeruli in each group, and the equation GV = (β/κ) × GA3/2 (1), where β = 1.38, the shape coefficient of spheres (the idealized shape of glomeruli), κ = 1.1, the size distribution coefficient, and GA is the glomerular area (7). Tubular injury was detected as tubular injury score by using a method described previously (19, 20). The scoring criteria for staining results were as follows: Score 0, no tubular injury; Score 1, < 10% of tubules injured; Score 2, 10–25% of tubules injured; Score 3, 26–50% of tubules injured; Score 4, 51–75% of tubules injured; Score 5, > 75% of tubules injured.

### ELISA

Renal tissues levels of mouse IL-17A, IFN-γ (MULTISCIENCES: EK217/2-AW1, EK280/3-AW1), IL-6, IL-4 and IL-10 (Elabscience: E-EL-M0044c, E-EL-M0043c, E-EL-M0046c) were quantified by using an ELISA method according to the instructions. Renal tissue lysates were extracted according to the following formula: 50 mmol/L Tris–HCl, 0.2% Triton X-100, 2 mmol/L EDTA, 150 mol/LNaCl, 2 mmol/L EGTA, 0.3%IGEPAL, 10 μl/ml proteinase inhibitors cocktail, 10 μl/ml PMSF, and 10 μl/ml orthovanadate (21).

### Flow cytometry and isolation of single-cell suspensions from the kidney

FACS analysis was performed according to the previous reports (22). After perfusion of the kidneys with cold 1 × PBS, kidneys were removed, cut into fragments, and digested with 1 mg/ml collagenase and 0.1 mg/ml DNAase for 1 h at 37°C with intermittent agitation. Kidney fragments were passed through a 70-μm mesh (FALCON, 352350), producing single-cell suspensions. Approximately 1 × 10^6^ cells were stained for 30 min at room temperature with antibodies including anti-Mouse CD45-PE (8205729; Invitrogen), anti-CD11b-APC (8278517; BD Pharmingen), and anti-Mouse CD206-PE (4336334; BD Pharmingen), and resuspended in 1 × PBS. The suspensions were washed thrice with 1 × PBS, resuspended in 1 × PBS, and analyzed on High configuration Analysis Flow Cytometry (BECKMAN, CytoFLEX LX).

### Statistics

GraphPad Prism 8.0 (provided by La Jolla, CA, USA) was used for statistical analyses, and the values were expressed in the form of mean ± SD. Two groups with the data obeying normal distribution were compared by *t*-test, and Analysis of Variance (ANOVA) along with Tukey’s test was used for the comparison among several groups. *P* < 0.05 was considered statistically significant. “* means *P* < 0.05,” “^**^ means *P* < 0.01,” “^***^ means *P* < 0.001.”

## Results

### TKIs promotes the expression of RelA in glomeruli and induces proteinuria

**FIGURE 1:**
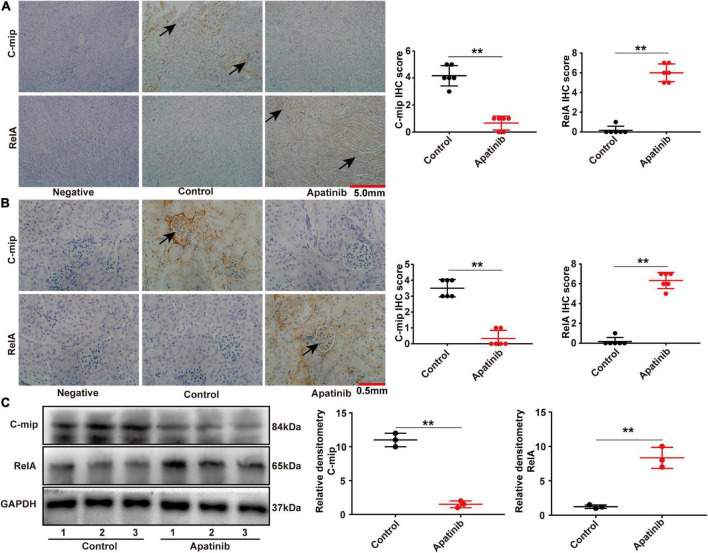
TKIs promotes the expression of RelA in glomeruli and tubules. **(A,B)** Representative images of immunohistochemistry and staining scores of RelA and c-mip in the kidney tissues of mice (*n* = 6, each group). **(C)** RelA and c-mip in mouse kidneys analyzed by Western blotting (*n* = 3, each group), **p < 0.01.

Our previous study found that TKIs treatment induced two kinds of glomerular damage, TMA and FSGS. In normal human kidneys, RelA was only expressed in podocytes, whereas c-mip was scarcely, or not, detected in human kidneys. RelA was highly expressed in glomerular endothelial cells and podocytes in TMA patients, but c-mip was not detected. By contrast, the relative abundance of c-mip was greatly increased in FSGS, whereas it was rarely detected in TMA (13). In order to observe whether TKIs influence renal RelA pathway, we sacrificed apatinib-treated mice and compared the kidneys with control. Immunohistochemical staining showed that apatinib increased the expression of RelA and inhibited the expression of c-mip in C57BL/6 mice kidneys, as well as glomeruli (Figures 1A, B). These results were also confirmed by Western-blot (Figure 1C).

### TKIs treatment causes podocyte loss and foot process effacement

**FIGURE 2:**
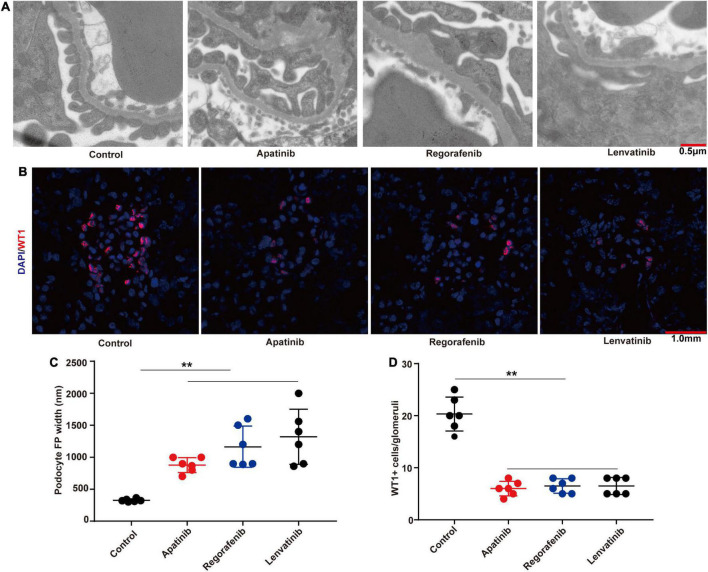
TKIs treatment causes podocyte loss and foot process effacement. **(A)** Representative glomerular filtration barrier of mice treated with apatinib, regorafenib, and lenvatinib under a transmission electron microscope (*n* = 6, each group). **(B)** Representative glomerular WT-1 staining of mice treated with apatinib, regorafenib, and lenvatinib. 12 fields were randomly selected for WT-1 + staining quantification (*n* = 6 in each group). **(C)** The distribution width of podocytes in each group. **(D)** WT-1 expression in each group, **p < 0.01.

The tumor-bearing C57BL/6 mice were treated by three TKIs (apatinib, regorafenib, and lenvatinib) to establish models of kidney injury. Through transmission electron microscopy, we found that TKIs mainly led to the loss and foot process effacement of podocytes (Figure 2A), the fluorescence intensity of the WT-1 (a podocyte-specific marker) in kidneys treated with apatinib, regorafenib, and lenvatinib was significantly lower than that in the control group (Figure 2B). The width of podocyte foot process and the quantitative analysis of the ratio of WT-1 positive cells to glomerular podocytes also confirmed these findings (Figures 2C, D).

### LDP relieves TKIs-induced proteinuria by inhibiting the expression of RelA in renal tissues

**FIGURE 3:**
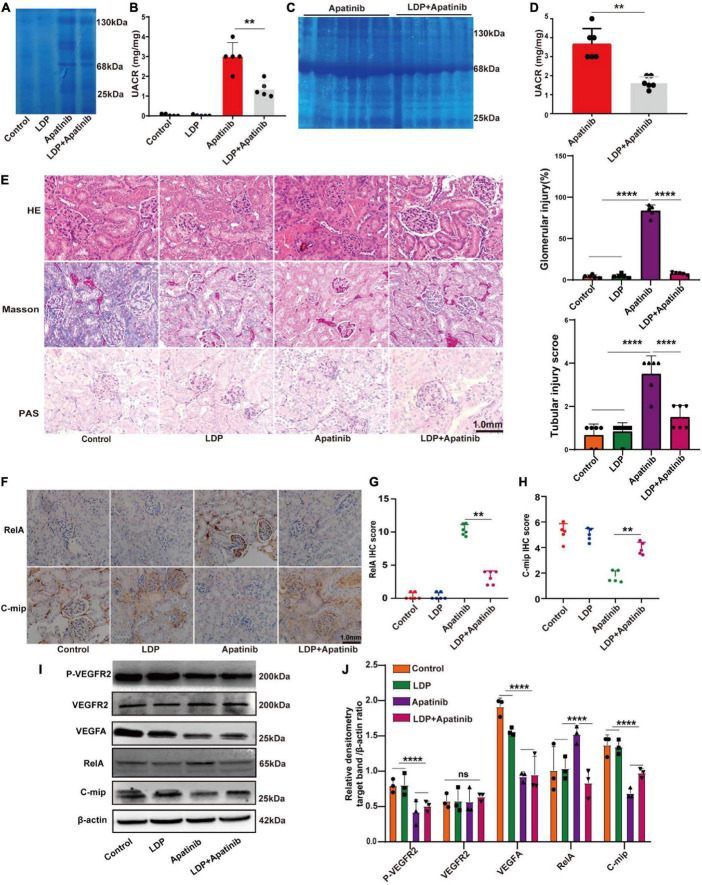
LDP relieves TKIs-induced kidney damage by inhibiting the expression of RelA in kidneys. **(A)** Albuminuria (10 μl urine in each lane) in male BALB/c nude mice treated with LDP, apatinib and LDP + apatinib in Coomassie gel (*n* = 8). **(C)** Proteinuria (10 μl urine in each lane) in male C57BL/6 mice treated with apatinib in Coomassie gel (*n* = 8). Tubular injury, glomerular injury and severe kidney injury representatively proteins of mice were treated with LDP, apatinib and LDP + apatinib, or apatinib alone (25, 68, and 130 kDa). **(B,D)** Quantitative analysis of albumin/creatinine (UACR) in urine (8 mice in each group). **(E)** Typical HE staining, PAS staining and Masson staining of renal tissues and staining scores of Glomerular injury and Tubular injury in mice. **(F–H)** Representative images of immunohistochemistry and staining scores of RelA and c-mip in the kidney cortex tissues of mice (*n* = 6 in each group). **(I,J)** Western blotting analysis of P-VEGFR2, VEGFR2, VEGFA, RelA and c-mip in mouse kidneys (*n* = 3 samples per group), **p < 0.01, ****P < 0.0001, ns represents no significant difference.

The effect of LDP on TKIs-induced proteinuria was next investigated. BALB/c nude mice were divided into four groups and treated with vehicle, LDP, TKIs, and TKIs + LDP, respectively. Coomassie brilliant blue staining was used for qualitative analysis (Figures 3A, C) of proteinuria levels in each group, and the ratio of urine protein to urine creatinine was used for quantitative analysis of proteinuria in each group (Figures 3B, D). The results showed that LDP alone had no effect on normal kidneys, while the proteinuria level of TKIs + LDP group was significantly lower than that of TKIs alone group. Histopathological staining showed that LDP attenuated glomerular injury induced by TKIs (Figure 3E). Western-blot and immunohistochemical data showed that LDP reduced the expression of RelA in TKIs-treated kidneys but did not affect the inhibitory effect of TKIs on VEGF signaling pathway (Figures 3F–J). These results indicated that LDP alleviates TKIs-induced proteinuria by inhibiting RelA in kidneys.

### Apatinib increases the expression of inflammatory factors and reduces the infiltration of M2 macrophages in the kidney

**FIGURE 4:**
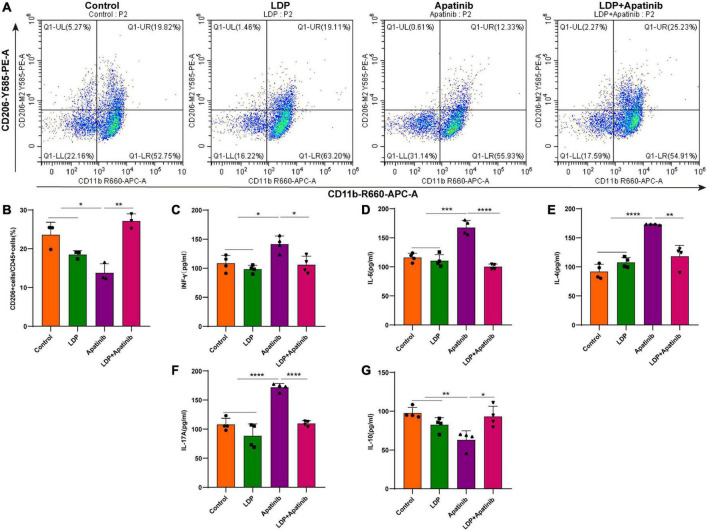
*In vivo* Apatinib increases renal expression of pro-inflammatory factors and reduces the infiltration of M2 macrophages. **(A,B)** FACS analysis showed that macrophage M2 polarization for macrophages from control, LDP, apatinib, LDP + apatinib kidneys and quantitative analysis of four animals per group. **(C–G)** In renal extracts, cytokine levels were evaluated by ELISA of four animals per group. *p < 0.05, **p < 0.01, ***p < 0.001, and ****P < 0.0001.

The macrophage is a major regulator in the formation of the inflammatory microenvironment. RelA plays a vital role in promoting the classical activation of macrophages (23). M2 macrophages are important anti-inflammatory immune cells in the renal microenvironment, we then measured the accumulation and activation of infiltrating M2 macrophages in the apatinib-induced inflammatory injury kidneys. To examine the infiltration of M2 macrophages, single-cell suspensions were prepared from kidneys of control, LDP, apatinib and LDP + apatinib mice and parsed via flow cytometry to isolate CD11b^+^CD206^+^macrophages (Figure 4A). Compared with control and LDP mice, kidneys from apatinib mice contained lower numbers of CD11b^+^CD206^+^macrophages. In addition, treatment with LDP + apatinib ameliorated apatinib-induced renal damage and markedly increased the presence of infiltrating CD11b^+^CD206^+^macrophages in renal tissues (Figure 4B). Under inflammatory conditions, some inflammatory factors produced by the kidney are regulated by RelA (21). Kidneys from apatinib-treated mice presented enhanced levels of the cytokines IFN-γ, IL-6, IL-4 and IL-17A compared with control and LDP mice, which were reduced by LDP + apatinib treatment (Figures 4C–F). By contrast, IL-10 was up-regulated by LDP + apatinib treatment (Figure 4G). In apatinib mice, the renal mRNA expression of the inflammatory factors IFN-γ, IL-6, IL-4 and IL-17A was significantly upregulated compared with control and LDP animals (real-time PCR, Supplementary Figures 2A–D), but IL-10 was down-regulated (Supplementary Figure 2E). Treatment with the LDP + apatinib restored IL-17A, IFN-γ, IL-6, IL-4 and IL-10 gene and protein expression to control levels (Supplementary Figures 2A–E). These data suggest that LDP alleviates apatinib induced renal inflammatory injury by increasing the infiltration of M2 macrophages and inhibiting the release of pro-inflammatory factors.

### LDP alleviates TKIs-induced proteinuria by improving podocyte loss and foot process effacement

**FIGURE 5:**
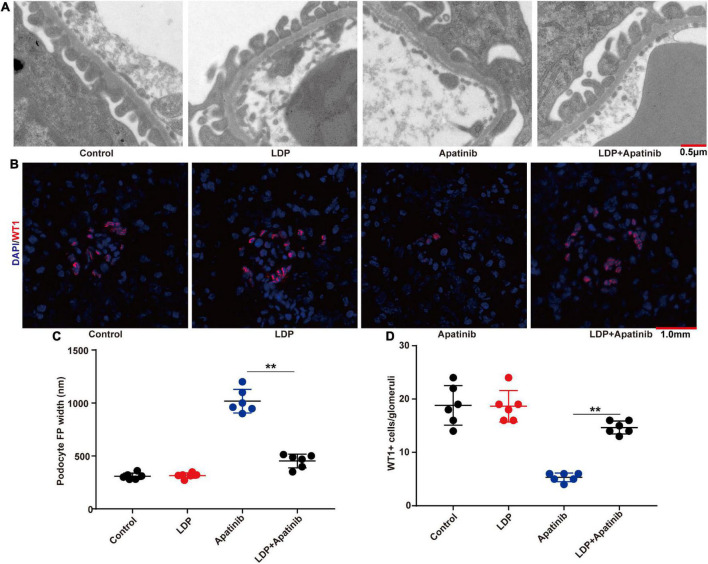
LDP alleviates podocyte loss and foot process effacement in BALB/c nude mice. **(A)** Representative glomerular filtration barrier of mice treated with LDP, apatinib and LDP + apatinib under a transmission electron microscope (*n* = 6 in each group). **(B)** Representative glomerular WT-1 staining of BALB/c nude mice which were treated with LDP alone, Apatinib alone, or both. 12 fields were selected randomly for WT-1 + staining quantification (*n* = 6 in each group). **(C)** The distribution width of podocytes in each group. **(D)** WT-1 expression in each group, **p < 0.01.

Given the strong relationship between podocyte injury and proteinuria, we next explored whether LDP alleviates proteinuria by acting on podocytes. The changes of podocytes in each group were compared, and it was found that LDP treatment ameliorated TKIs-induced podocyte loss and foot process effacement (Figure 5A). Compared with TKI alone group, there were a higher level of WT-1 protein (Figure 5B), a smaller width of podocyte foot process (Figure 5C), and a higher ratio of WT-1-positive cells to glomerular podocytes in LDP + TKI group (Figure 5D). Further, we discovered that Apatinib ultimately markedly caused renal cells apoptosis, compared with control and LDP mice. By contrast, treatment with LDP + apatinib reduced apatinib-induced renal cells apoptosis (Supplementary Figures 1A, B).

### LDP enhanced the anti-cancer effect of TKIs on C57BL/6 mice

**FIGURE 6:**
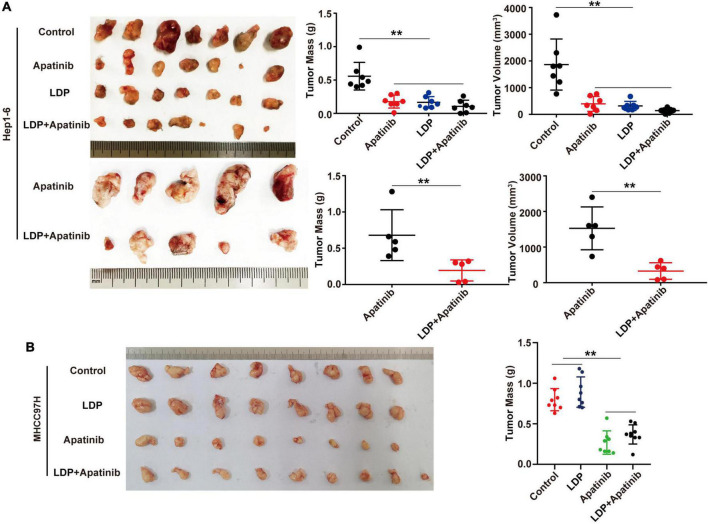
LDP enhances the anticancer effect of apatinib in immunogenic mice, but does not increase the anticancer effect of immunodeficient mice. **(A)** Subcutaneous tumors of C57BL/6 mice and volume and mass values at specific time points (*n* = 5 or 7). **(B)** Subcutaneous tumors of BALB/C mice and volume and mass values at specific time points (*n* = 8 or 9). **p < 0.01.

The previous study has reported that LDP can inhibit the development of gastric cancer. To evaluate the efficacy of LDP on HCC, C57BL/6 mice and BALB/C mice with HCC were used to establish the TKIs-induced kidney injury mouse model. Compared with TKIs alone group, the combination of LDP and TKIs significantly reduced the volume and weight of tumor in C57BL/6 mice (Figure 6A). However, there were no significant differences in tumor size and weight between BALB/C mice treated with TKIs alone and those treated with LDP + TKIs (Figure 6B), The biggest difference between C57BL/6 mice and BALB/C mice was the lack of cellular immunity in BALB/C mice, indicating that cellular immunity may play a role in LDP-mediated enhancement of the anti-cancer effect of TKIs on HCC.

## Discussion

Proteinuric kidney injury caused by TKIs and anti-VEGFA therapy was compared, and it was found that the glomerular lesions related to TKIs primarily involved podocytes. Although anti-VEGFA therapy primarily influenced endothelial cells, the injury of podocytes was actually the subordinate results (13). However, in an analysis of patients receiving antiangiogenic therapy, although no patients showed both FSGS-like lesions and TMA during TKIs or anti-VEGFA therapy (13), both lesions were associated with anti-VEGFA and TKIs therapy (13, 24), consistent with our previous findings. In normal kidneys, VEGFA is mainly produced by podocytes and acts on VEGFR2 on the surface of endothelial cells to maintain endothelial structure and function. Both TKIs and anti-VEGFA therapy block VEGFA/VEGFR2 signaling, but the subsequent renal pathological changes are not only endothelial cell damage, but also involve podocyte damages. These findings suggest that the kidney injury caused by VEGFA-VEGFR2 inhibitors is not derived from the injury of a single cell type, but mediated by the abnormal signaling crosstalk between various types of cells in the glomerulus.

Many studies have indicated the critical role of inflammation in proteinuria (25), but the mechanism has not been clearly elucidated. In addition to involvement in mammalian inflammation (26), the NF-κB family of transcription factors is also engaged in kidney injury (27), and plays a key role in renal inflammation (28). The inhibition of NF-κB activity has been shown to improve renal injury and proteinuria in animal models (26, 29, 30). However, whether NF-κB is involved in TKIs-induced proteinuric nephropathy remains unclear. Our study firstly found that TKIs led to increased expression of RelA in kidneys. The inhibition of RelA alleviated proteinuria, suggesting that RelA is critical for the development of TKIs-induced proteinuria. Podocyte injury has been reported to be directly associated with proteinuria (31), and can be induced by RelA-mediated inflammation (28, 31–33). In our study, TKIs led to podocyte loss and foot process effacement. There were a decreased level of WT-1 protein, an increased width of podocyte foot process, and a decreased ratio of WT-1-positive cells to glomerular podocytes in kidneys of TKIs-treated mice.

Traditional Chinese Medicine (TCM) is attracting increasing attention for its unique theory and long-term clinical practice (34). LDP is a TCM formula that can alleviate inflammation, oxidative stress and related kidney injury, and can also be used to treat DN (15). LDP has been reported to reduce testicular inflammation in aging rats by inhibiting the AMPK/Sirt1/NF-κB pathway (34). Given the performance of LDP in treating DN and reducing inflammation (15, 34), we empirically applied LDP in combination with apatinib to mice with HCC, and it was found that the combination significantly inhibited the activity of RelA and alleviated the proteinuria induced by apatinib alone. LDP can inhibit the development of gastric cancer (15). Therefore, the effect of LDP on the anti-cancer effect of apatinib was evaluated. The results demonstrated that LDP could synergize with the anti-cancer effect of apatinib in C57BL/6 mice, but not in BALB/C mice, suggesting that LDP may exert its anti-cancer effect through T cells.

Our study showed that LDP can significantly relieve TKIs-induced proteinuria with few side effects. However, there are still several challenges for the clinical application of LDP in the treatment of TKI-induced proteinuria. Firstly, the efficacy and safety of LDP in the treatment of TKI-induced proteinuria remain to be clinically verified. Secondly, the specific effective component of LDP that works in alleviation of proteinuria is still unclear due to poor understanding of the components of LDP. Fortunately, the development of new smart devices and modern technologies such as cell biology will accelerate exploration of the specific mechanism of LDP alleviating TKIs-induced proteinuria, and will further promote our understanding about TCM.

## Conclusion

This study demonstrated that LDP attenuated the inflammatory injury of podocytes through inhibiting activation of RelA, and subsequently relieved TKI-related proteinuria with few side effects. Besides, LDP improved the anti-tumor activity of apatinib in tumor-bearing mice, in which the immune system may play an important role. In summary, the therapeutical effects of LDP on TKI-related proteinuria provided a scientific basis for their compatibility in TCM prescriptions.

## Data availability statement

The original contributions presented in this study are included in the article/Supplementary material, further inquiries can be directed to the corresponding authors.

## Ethics statement

The animal study was reviewed and approved by Tianjin Medical University Cancer Institute and Hospital. Written informed consent was obtained from the owners for the participation of their animals in this study.

## Author contributions

ZF: writing – original draft and writing – review and editing. SZ: methodology, development, or design of methodology. XG: data curation. TG: formal analysis. CW: visualization. JZ: investigation. YW: software. HG: supervision. LW: validation. TZ: conceptualization, project administration, funding acquisition, and management and coordination responsibility for the research activity planning and execution. All authors contributed to the article and approved the submitted version.
