# Supplementary material for: LDP alleviates TKI-induced proteinuria through reversing the expression of RelA in renal tissues
Source: Front Med (Lausanne). 2023 Jan 19;10:1095344. doi: 10.3389/fmed.2023.1095344 (PMC9892181; doi:10.3389/fmed.2023.1095344)
Supplement: Supplementary file 1 [file Data_Sheet_1.docx]

Supplementary Material

##
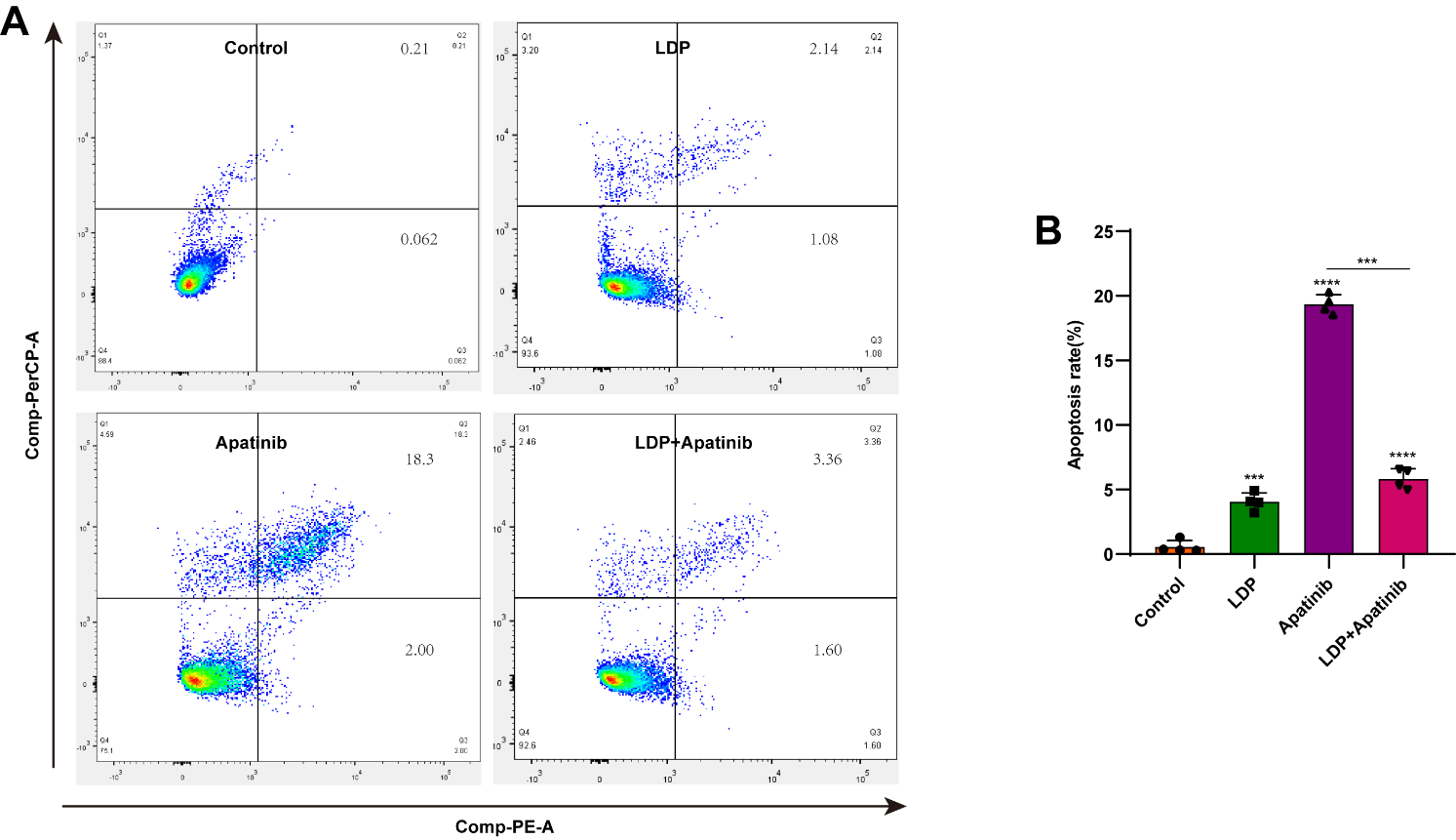


**Supplementary Figure1.** In vivo Apatinib increases renal cells apoptosis. (A,B) In renal extracts, FACS analysis showed that renal cells apoptosis from control, LDP, apatinib, LDP+apatinib kidneys and quantitative analysis of four animals per group.


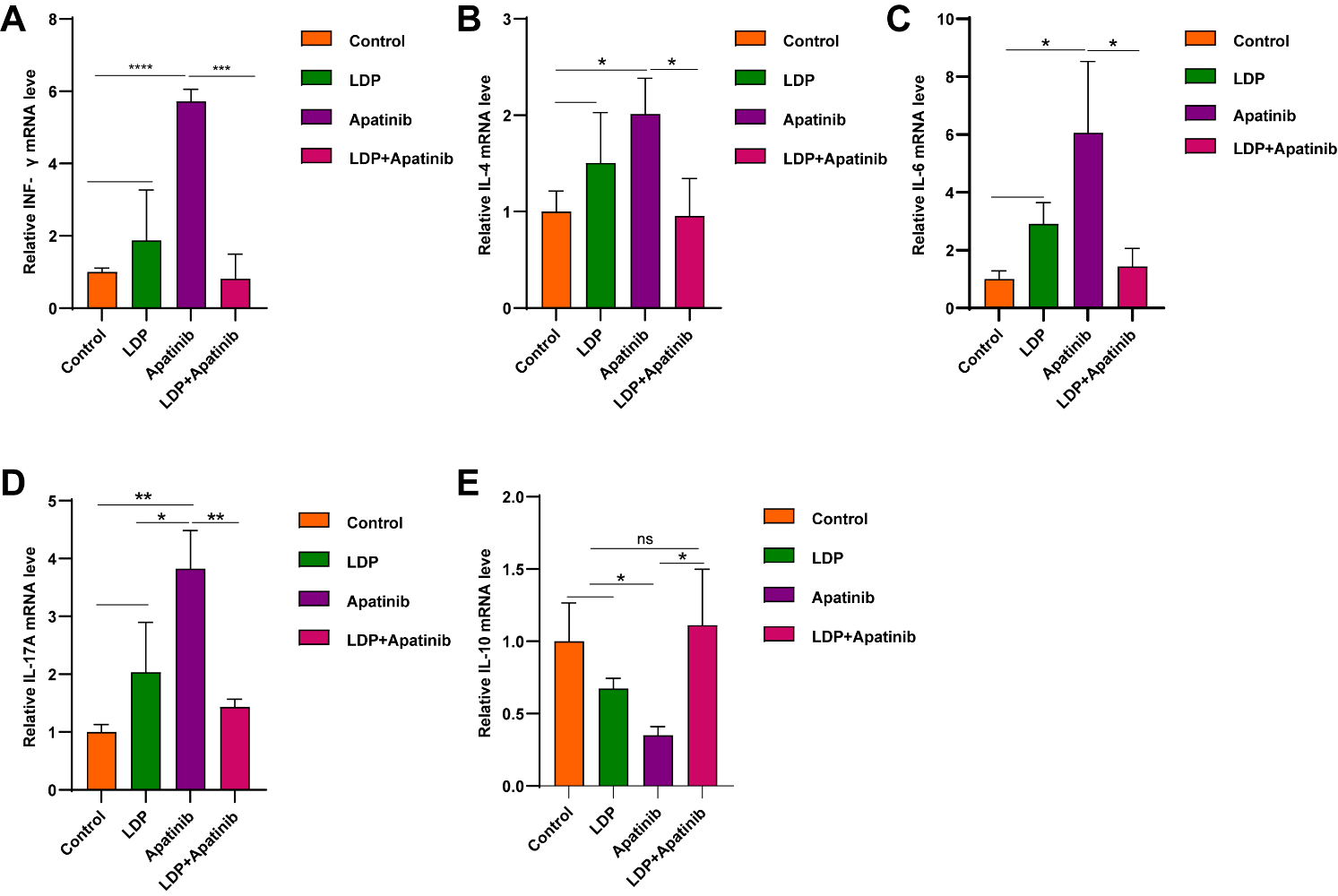


**Supplementary Figure2.** Apatinib increases the mRNA expression of inflammatory factors. (A,B,C,D,E) IFN-γ, IL-4, IL-6, IL-17A and IL-10 gene expression as mean±SEM of three animals per group analyzed by real-time PCR.
